# Assumptions about fence permeability influence density estimates for brown hyaenas across South Africa

**DOI:** 10.1038/s41598-020-77188-7

**Published:** 2021-01-12

**Authors:** Kathryn S. Williams, Samual T. Williams, Rebecca J. Welch, Courtney J. Marneweck, Gareth K. H. Mann, Ross T. Pitman, Gareth Whittington-Jones, Guy A. Balme, Daniel M. Parker, Russell A. Hill

**Affiliations:** 1 Department of Anthropology, Durham University, Dawson Building, South Road, Durham, DH1 3LE UK; 2 Primate and Predator Project, PO Box 522, Louis Trichardt, 0920 South Africa; 3 Department of Zoology, University of Venda, Thohoyandou, 0950 South Africa; 4 Institute for Globally Distributed Open Research and Education (IGDORE), Johannesburg, South Africa; 5 School of Biology and Environmental Sciences, University of Mpumalanga, Nelspruit, 1200 South Africa; 6 Wildlife and Reserve Management Research Group, Department of Zoology and Entomology, Rhodes University, PO Box 94, Grahamstown, 6140 South Africa; 7 Panthera, 8 W 40th Street 18th Floor, New York, NY 10018 USA; 8 Institute for Communities and Wildlife in Africa, University of Cape Town, Private Bag X3, Rondebosch, 7701 South Africa

**Keywords:** Conservation biology, Zoology

## Abstract

Wildlife population density estimates provide information on the number of individuals in an area and influence conservation management decisions. Thus, accuracy is vital. A dominant feature in many landscapes globally is fencing, yet the implications of fence permeability on density estimation using spatial capture-recapture modelling are seldom considered. We used camera trap data from 15 fenced reserves across South Africa to examine the density of brown hyaenas (*Parahyaena brunnea*). We estimated density and modelled its relationship with a suite of covariates when fenced reserve boundaries were assumed to be permeable or impermeable to hyaena movements. The best performing models were those that included only the influence of study site on both hyaena density and detection probability, regardless of assumptions of fence permeability. When fences were considered impermeable, densities ranged from 2.55 to 15.06 animals per 100 km^2^, but when fences were considered permeable, density estimates were on average 9.52 times lower (from 0.17 to 1.59 animals per 100 km^2^). Fence permeability should therefore be an essential consideration when estimating density, especially since density results can considerably influence wildlife management decisions. In the absence of strong evidence to the contrary, future studies in fenced areas should assume some degree of permeability in order to avoid overestimating population density.

## Introduction

Natural barriers such as bodies of water and mountain ranges influence movement patterns and gene flow in wildlife populations^1–3^. Man-made physical barriers such as roads and fences similarly impact wildlife^4–7^. With the human population undergoing exponential growth and a continuing demand for ecotourism, enclosing wildlife in fenced areas is widespread in southern Africa^8,9^. Fences demarcate boundaries, reduce human-wildlife conflict, protect resources, and prevent disease transmission; yet they also disrupt the natural movement patterns of animals, which can cause ecosystem imbalances such as reducing genetic influx and causing over-exploitation of resources^8,10^. As a result, fencing for conservation is highly controversial^8,11^.

 In many parts of the world, wildlife is confined to fenced areas, and this is especially true in South Africa where it is a legal requirement to fence an area containing dangerous game species^8,12^. Although fences are often successful at confining cattle and large herbivores, fences are semi-permeable for many mammals including some large predators^13–15^. Holes under fences are created by erosion and by digging species^16^, and when a fence line is breached, species detect and exploit holes quickly^17^. To maintain the integrity of a fence, continuous upkeep is required which is costly (approximately US $32,000 per annum to maintain 100 km of fencing in South Africa)^16^. Maintenance of the majority of fences is underfunded in Africa, leading to a high occurrence of boundaries that are semi-permeable for species such as mammalian carnivores^10^.

In reserves where fences, including predator proof fences, are not maintained to the highest standard, brown hyaenas (*Parahyaena brunnea*) transverse boundaries by digging new holes and opportunistically expanding pre-existing holes^18^. Although brown hyaenas can survive, and often succeed, outside of protected areas, the highest population densities of brown hyaenas have been reported in small fenced reserves that are thought to be impermeable to the movement of brown hyaenas^19–21^. It is speculated that these brown hyaena population densities resulted from a lack of emigration, small reserve size, an abundance of large sympatric predators (carrion providers), and high levels of prey availability^19,21^. The influence of these factors on hyaena population density (the number of individuals per unit area), however, has not been tested.

Spatial capture-recapture (SCR) is a common method utilised to estimate densities from camera trap images^22^. The state variable in SCR is a spatial point process where each point represents an individual’s activity centre, and the state-space incorporates these regions of activity centres^23^. The hypothesis being that animals with activity centres outside of this state-space region have little chance of being captured^23^. One is free to specify the state-space and therefore estimates will be biased if this area is so small that some captured animals have activity centres outside of this region. In enclosed areas, some researchers restrict the state-space to fence lines when estimating population densities of large carnivores using SCR modelling^19,21,24,25^. In open areas or fenced areas where cross-boundary movement is probable, a larger state-space buffer is employed in SCR modelling to encompass home ranges and activity centres that span beyond the study area^26,27^. How one deals with individuals that do not necessarily reside within a reserve but do traverse, and are detected, within it (i.e. the definition of the state-space), therefore has strong implications for the density estimates produced. The accuracy of density estimates is paramount as they are often central to conservation management decisions, yet there is a paucity of research on how assumptions regarding the permeability of fenced reserve boundaries (i.e. the defined state-space) affects density estimates, especially for large carnivores.

Due to their small population size and high levels of intentional and accidental persecution, brown hyaenas are listed as Near Threatened both globally^28^ and regionally in South Africa^29^. Their current resident range is restricted to six countries including South Africa, which is thought to support approximately 20% of the total remaining population^29^. Furthermore, research into the distribution, population size, and trends of brown hyaenas at a national scale has been identified as a top priority for brown hyaena conservation^28,29^, requiring reliable density estimates over a large area. In this study, we estimate the population density of brown hyaenas in 15 fenced reserves in South Africa, making two contrasting assumptions about the permeability of the reserve boundary fences to the movement of brown hyaenas at each site: 1) reserve boundaries are impermeable (with the state-space clipped to the reserve boundary fence line), and 2) reserve boundaries are permeable (with a larger, unclipped state-space). We consider the repercussions of varying state-space in SCR modelling and the implications for conservation management when assessing fenced areas. In addition, we investigate which factors drive brown hyaena density at fenced sites and compare the results when fences are considered impermeable and permeable.

## Results

**Figure 1:**
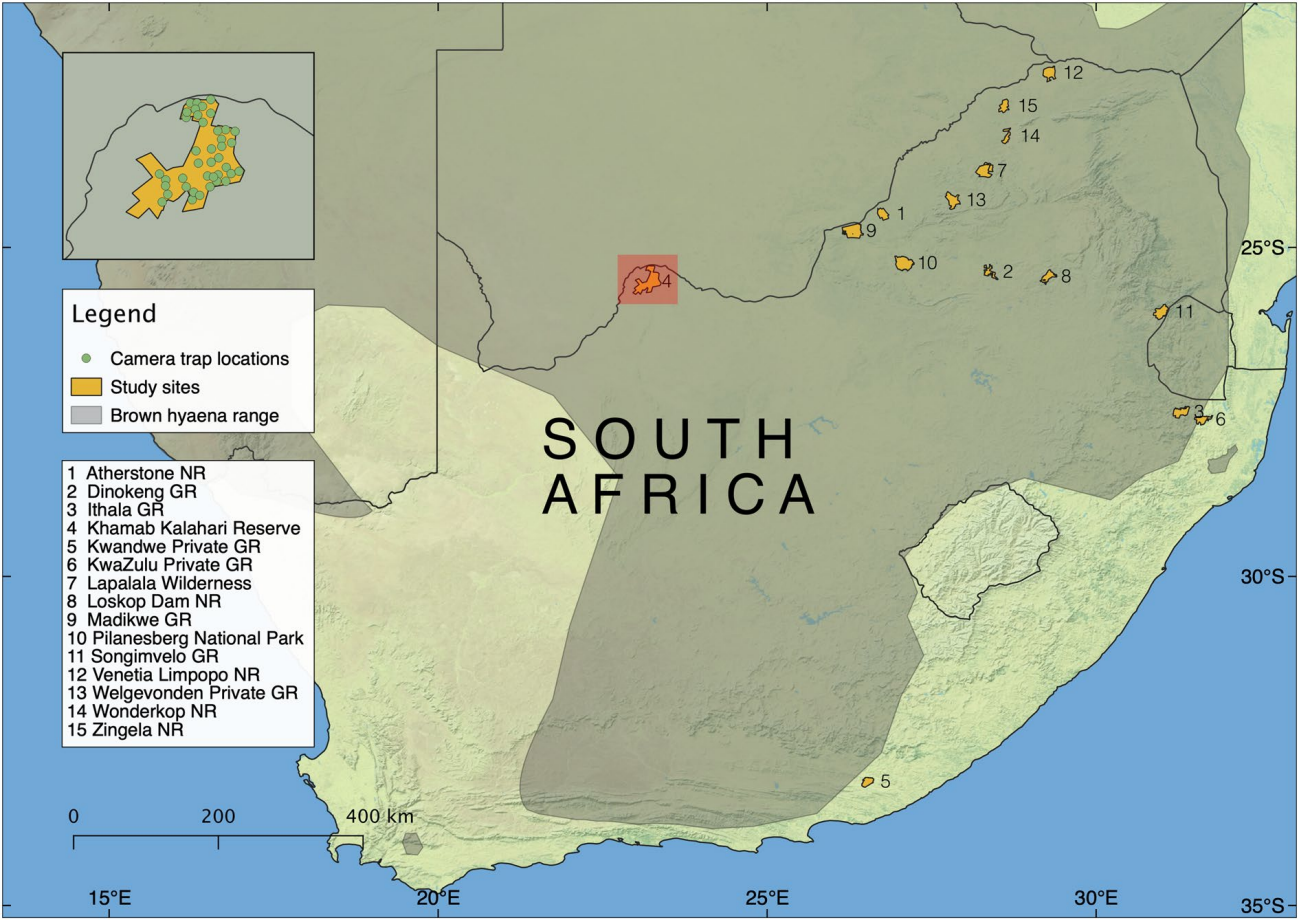
Map showing the locations of the survey sites where brown hyaena population densities were estimated. Inset map shows an example layout of a camera trap array at Khamab Kalahari Reserve. In the legend NR refers to Nature Reserve, and GR refers to Game Reserve. Created using QGIS 3.10.10^30^, using hyaena range data from^28^ and base map data from^31^.

At 15 survey sites across South Africa (Fig. 1, Table 1) we collected 2690 camera trapping capture events of brown hyaenas (Table S1). We discarded 298 (11.08%) brown hyaena captures because image quality was insufficient to allow identification of individuals (Table S1). The majority of discarded captures had only one photograph (n = 289); therefore, the hyaena was only visible from one side, making identification more difficult. A total of 362 identifiable brown hyaenas were captured on 2392 occasions (Table S1).

**Table 1:** Camera trap surveys in South Africa. Camera area is the minimum convex polygon area covered by camera trap stations. Survey effort is the total number of trap nights each camera survey was active. Camera area is the minimum convex polygon of the camera trap array.

Survey site	Reserve size (km^2^)	Minimum convex polygon of camera trap area (km^2^)	Survey period	Camera stations	Survey effort (trap nights)
Atherstone Nature Reserve	240	180	Oct 2016–Dec 2016	36	1797
Dinokeng Game Reserve	185	173	Jul 2016–Aug 2016	36	1586
Ithala Game Reserve	296	236	July 2016–Sep 2016	30	1315
Khamab Kalahari Reserve	955	570	Aug 2016–Oct 2016	37	1744
Kwandwe Private Game Reserve	183	135	Mar 2017–Apr 2017	40	1860
KwaZulu Private Game Reserve	185	135	Nov 2015–Dec 2015	34	2645
Lapalala Wilderness	360	331	Oct 2016–Dec 2016	39	1932
Loskop Dam Nature Reserve	232	170	Oct 2016–Dec 2016	34	1774
Madikwe Game Reserve	600	306	Nov 2016–Dec 2016	36	1472
Pilanesberg National Park	550	247	Mar 2016–Apr 2016	40	1785
Songimvelo Game Reserve	490	112	Mar 2016–Apr 2016	27	1127
Venetia Limpopo Nature Reserve	316	237	Jul 2016–Aug 2016	39	1934
Welgevonden Private Game Reserve	375	203	Apr 2016–May 2016	40	1292
Wonderkop Nature Reserve	160	150	Jan 2015–Mar 2015	37	1579
Zingela Nature Reserve	219	177	May 2016–Jun 2016	39	1690

**Figure 2:**
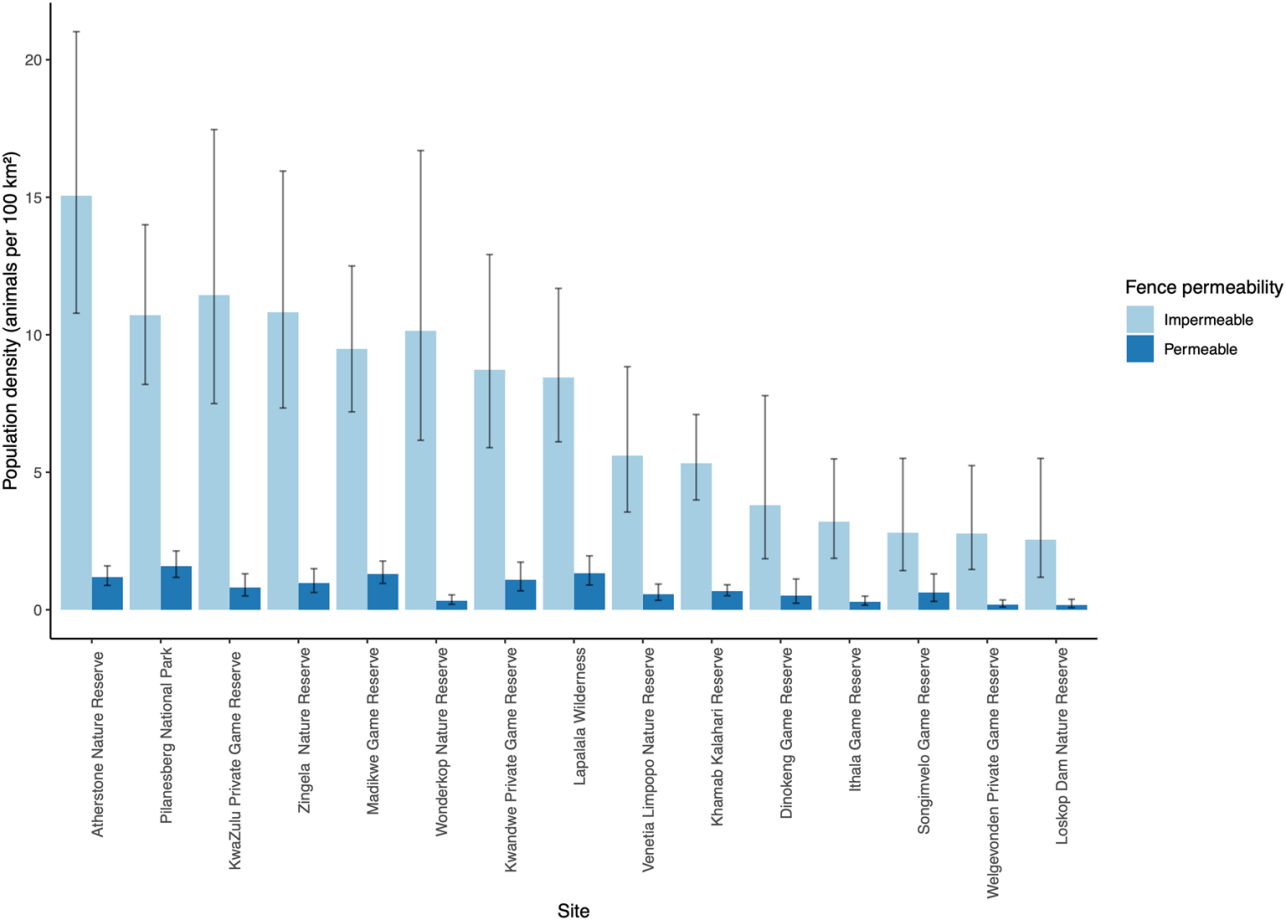
Population density estimates for brown hyaenas as each site assuming that fences were either permeable or impermeable to hyaena movement. Error bars show 95% confidence intervals.

The top population density models relating to both assumptions of fence permeability included only the site covariate on both *g0* (detection probability when the distance between the activity centre of an animal and the camera trap is zero) and on density (Table 2, Table S2). Density estimates derived from the top model ranged from 2.55 to 15.06 animals per 100 km^2^ at each site when fences were considered to be impermeable, and from 0.17 to 1.59 animals per 100 km^2^ when fences were considered to be permeable to hyaena movement (Fig. 2, Table S3).

**Table 2:** Comparison of models of *g0* and density fitted to camera trap data of brown hyaena captures across South Africa. Parameters include: D (density estimate) and *g0* (baseline detection). Covariates include: b (learned response; *g0* only); session (site), LeopardRAI (relative abundance index (RAI) of leopards), SpHyRAI (RAI of spotted hyaenas), HumanRAI (RAI of humans), HumanDensity (population density of humans) PreyRAI (RAI of prey species), ReserveSize (size of reserve). All models utilised the hazard rate detection function. Models shown in bold are the top models within each subset.

Fence permeability	Model	Number of parameters	logLik	AICc	ΔAICc	AICcwt
Impermeable	**g0 ~ session**	**17**	**− 13,583.25258**	**27,202.264**	**0**	**1**
	D ~ 1 g0 ~ ReserveSize	5	− 13,726.6259	27,463.418	261.154	0
	g0 ~ PreyRAI	4	− 13,728.84766	27,465.806	263.542	0
	g0 ~ HumanRAI	4	− 13,731.93908	27,471.989	269.725	0
	D ~ 1 g0 ~ SpHyRAI	5	− 13,780.73571	27,571.638	369.374	0
	D ~ 1 g0 ~ LeopardRAI	5	− 13,789.21782	27,588.602	386.338	0
	D ~ 1 g0 ~ HumanDensity	5	− 13,790.37303	27,590.913	388.649	0
	D ~ 1 g0 ~ 1	4	− 13,811.65901	27,631.429	429.165	0
	D ~ 1 g0 ~ b	5	− 13,810.74387	27,631.654	429.39	0
	**D ~ session g0 ~ session**	**32**	**− 13,619.62238**	**27,309.587**	**0**	**1**
	D ~ HumanRAI g0 ~ session	19	− 13,656.18928	27,352.575	42.988	0
	D ~ 1 g0 ~ session	18	− 13,662.96435	27,363.9	54.313	0
	D ~ SpHyRAI g0 ~ session	19	− 13,662.189	27,364.575	54.988	0
	D ~ PreyRAI g0 ~ session	19	− 13,662.4187	27,365.034	55.447	0
	D ~ LeopardRAI g0 ~ session	19	− 13,662.96355	27,366.124	56.537	0
	D ~ HumanDensity g0 ~ session	19	− 13,663.26425	27,366.725	57.138	0
	D ~ ReserveSize g0 ~ session	19	− 13,667.47856	27,375.154	65.567	0
Permeable	**D ~ 1 g0 ~ session**	**18**	**− 13,758.6739**	**27,555.319**	**0**	**0.9571**
	D ~ 1 g0 ~ SpHyRAI	5	− 13,775.68221	27,561.531	6.212	0.0429
	D ~ 1 g0 ~ HumanRAI	5	− 13,784.58262	27,579.332	24.013	0
	D ~ 1 g0 ~ LeopardRAI	5	− 13,786.04496	27,582.257	26.938	0
	D ~ 1 g0 ~ ReserveSize	5	− 13,788.84003	27,588.547	33.228	0
	D ~ 1 g0 ~ HumanDensity	5	− 13,799.30979	27,608.786	53.467	0
	D ~ 1 g0 ~ b	5	− 13,803.15885	27,616.484	61.165	0
	D ~ 1 g0 ~ 1	4	− 13,807.76959	27,623.65	68.331	0
	D ~ 1 g0 ~ PreyRAI	5	− 13,807.89884	27,625.964	70.645	0
	**D ~ session g0 ~ session**	**32**	**− 13,700.353**	**27,471.049**	**0.00**	**1**
	D ~ PreyRAI g0 ~ session	19	− 13,755.963	27,552.123	81.07	0
	D ~ SpHyRAI g0 ~ session	19	− 13,756.577	27,553.35	82.30	0
	D ~ HumanRAI g0 ~ session	19	− 13,756.795	27,553.786	82.74	0
	D ~ 1 g0 ~ session	20	− 13,758.674	27,555.319	84.27	0
	D ~ LeopardRAI g0 ~ session	19	− 13,758.376	27,556.948	85.90	0
	D ~ HumanDensity g0 ~ session	19	− 13,759.67107	27,559.539	88.49	0
	D ~ ReserveSize g0 ~ session	19	− 13,763.359	27,566.915	95.87	0

**Figure 3:**
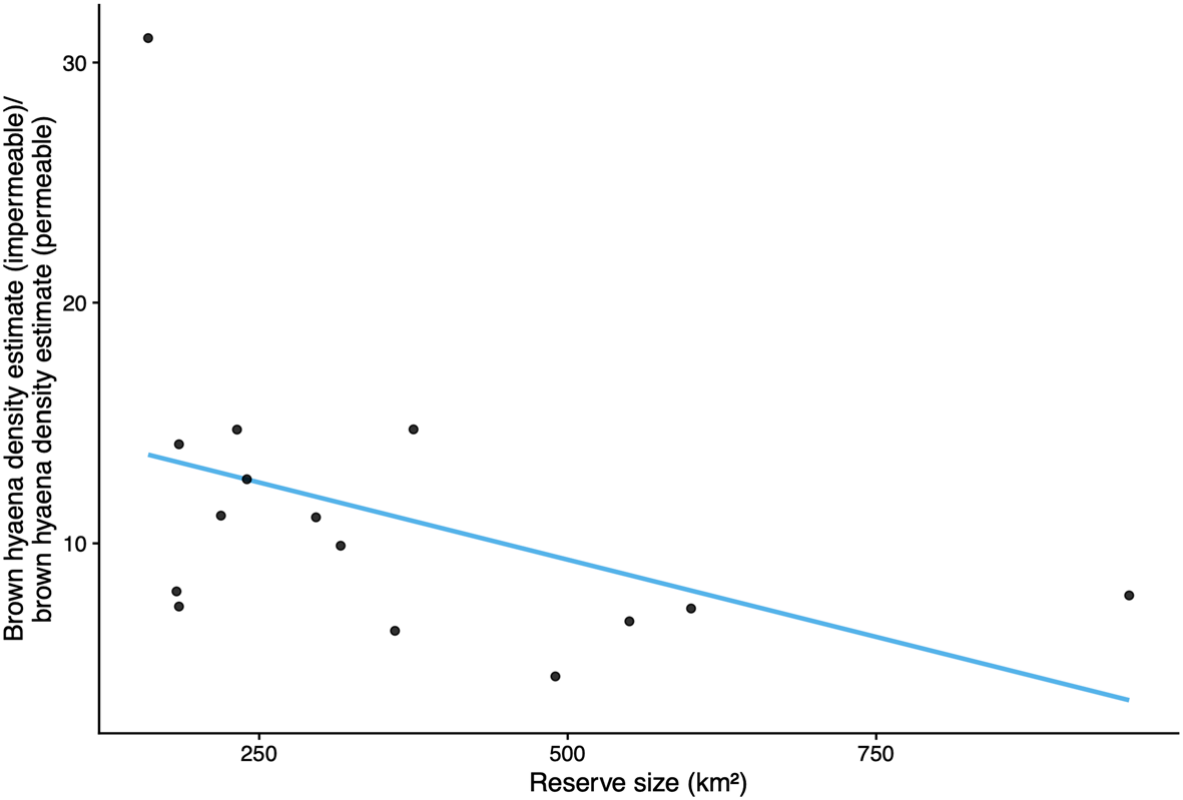
Relationship between reserve size and the ratio of brown hyaena population density estimates when using differing assumptions of fence permeability.

When fences were assumed to be permeable, hyaena density estimates were on average 9.5 times lower than when fences were assumed to be impermeable to hyaena movement. Furthermore, we found an inverse relationship between reserve size and the ratio of brown hyaena density estimates modelled using different assumptions about fence permeability (Fig. 3, Table 3). In contrast, population size estimates were 1.6 times greater for models that assumed fence permeability (see Table S4). The general patterns of activity centre location were relatively similar for both model permeable and impermeable formulations at most study sites (Fig. S1). Estimates in smaller reserves were more sensitive to assumptions regarding the permeability of reserve boundaries to brown hyaena movement.

**Table 3:** Comparison of fit between models of the relationship between reserve size and the ratio of brown hyaena density estimated using differing assumptions of fence permeability.

Distribution	AIC	dAIC	Degrees of freedom
Inverse Gaussian	87.0	0.0	3
Gamma	89.6	2.6	3
Null model	91.9	4.9	2
Gaussian	100.0	13.0	3

## Discussion

Our study represents the largest and widest-ranging collection of density estimates to date for brown hyaenas. Our density estimates for each site varied substantially depending on whether fences were considered to be permeable or impermeable to the movement of brown hyaenas. Hyaena population densities were approximately ten times greater among impermeable estimates than permeable estimates due to the state-space difference associated with this assumption.

The population density of brown hyaenas has been estimated at only a handful of sites that are fully enclosed by fencing^19,21,32,33^, despite fences encompassing a large proportion of protected and non-protected land throughout the species range^8,9,34^. Overall, our estimates fit within the ranges of most previous studies^20,21,35,36^. Our estimates for permeable fenced areas (0.17–1.59 animals per 100 km^2^) were slightly lower than, but comparable with, the few available previous brown hyaena density estimates in permeable fenced reserves (approximately 3 animals per 100 km^2^^32,33^). When fences were assumed to be impermeable, our brown hyaena density estimates (2.55-15.06 animals per 100 km^2^) were also comparable to the high density estimates calculated using SCR in fenced areas assumed to be impermeable to hyaena movements (15^21^ to 24.01^19^ brown hyaenas per 100 km^2^).

Many studies do not restrict the state-space to fence lines and reserve boundaries, resulting in relatively low density estimates^22,37–39^. Less commonly the state-space is restricted to fences, often resulting in record high densities^19,21,24^. Our results show that the corresponding change in the state-space buffer used in SCR analysis when fences are assumed to be permeable or impermeable to animal movements results in substantially different densities, even when using the same capture histories, which may have important repercussions with regard to carnivore management objectives.

We found that the implications of truncating the state-space to the fence line are area dependant, where smaller reserves are more sensitive to assumptions regarding fence permeability than larger reserves. In larger areas, the trapping grid is likely located further away from the fence line, and thus density estimates are less sensitive to the state-space defined because the individuals exposed to sampling in these areas should be mostly those that have their activity centres within the reserve. In contrast, in smaller areas the trapping grid is likely located closer to the fence line and thus the individuals exposed to sampling in these areas are more likely to hold activity centres outside of the reserve. Consequently, assuming fences are impermeable (i.e. truncating the state-space) in a small, enclosed reserve would likely yield highly over-estimated densities. This indicates that decisions regarding fence permeability should not be taken lightly when studying less extensive reserves or more fragmented habitats, which are relatively common in countries such as South Africa that make up a large proportion of the range of brown hyaenas^40,41^.

In addition, we suggest a more nuanced approach to defining habitat. Brown hyaenas are likely to be able to survive outside of protected areas across much of rural southern Africa. However, large carnivore densities are likely to be higher within protected areas^42^. This is not reflected in the estimates of population size, which assume equal habitat quality both within and outside of protected areas, and are thus likely to produce inflated population estimates when fences are assumed to be permeable. We suggest that future studies acknowledge this reality by including a measure of habitat quality into the state space mask used in SCR analyses^43,44^. This will allow researchers to account for the likely permeability of reserve fences, while simultaneously modelling the probable costs to individuals ranging outside of the reserve (lower habitat suitability, greater risk of persecution, etc.).

One of the main challenges of this study was the lack of reliable data on fence permeability and the extent of movement by brown hyaenas at each survey site. Due to the size of the reserves, the number of survey sites, and financial and temporal constraints, we were unable to accurately quantify the potential for brown hyaena movement through fences, thus leading us to examine two extremes of fence permeability. The assumption of complete permeability or impermeability is unlikely to be entirely accurate in the way predators use landscapes, and in reality, the permeability of many fences will likely lie somewhere between these extremes. For example African wild dogs (*Lycaon pictus*) can cross the “predator-proof” fence surrounding our Pilanesberg National Park study site^45^, so even predator-proof fencing often has some degree of permeability. Movement will be concentrated around holes in the fence line, thus creating spatial heterogeneity in fence permeability. Furthermore, as animals dig holes, fences are damaged or fall into disrepair, or fences are maintained, the degree of permeability will change dynamically over time, compounding the challenge of making objective and meaningful assessments of fence permeability. At present there is also no way of incorporating these data directly into available SCR models, but the development of such models would be one way to help calculate more accurate density estimates in these systems, should collection of fence quality data be possible. If fence holes are documented and remain persistent, they could be modelled using non-euclidean distance methods and integrated into SCR models (M. Efford, pers. comm.). The successful incorporation of permanent holes in density modelling will likely encourage methods to be devised that consider movement through ephemeral holes.

For both scenarios of fence permeability, the models with the most support included only the site covariate in models of both *g0* and hyaena population density. This suggests that site-specific factors were stronger determinants of brown hyaena abundance and detection probability than the other covariates included in the models. It was interesting that no support was found for an association between the relative abundance index (RAI) of competitor species and brown hyaena density, as variables were predictors of brown hyaena occupancy^46^. It is also possible that RAI lacks the precision to tease out these effects, and covariates such as absolute leopard (*Panthera pardus*) density may perform differently to leopard RAI, although these data are not yet available. Although RAI can be biased by ecological factors and sampling design^47^, numerous camera trapping studies use RAI as a proxy for covariates, especially when density estimates are unavailable^39,46,48^.

One potential caveat of the study is that camera trap spacing is a key element of SCR study design, so care should be taken to ensure that bycatch data are used appropriately. Our results could therefore be biased by estimating brown hyaena density using data that were collected using a design optimised for the estimation of leopard density, if leopards had much larger home ranges than brown hyaenas. But since brown hyaenas tend to have similar or larger home ranges than leopards^49–51^, we would expect the results to be comparable to a survey dedicated to brown hyaenas, and we would design the camera trap arrays in a very similar pattern for both species. Such a wide-ranging study using such a large dataset would not have been possible without using bycatch data.

## Conclusion

Assumptions regarding the permeability of fencing to the movement of brown hyaenas had a great influence on population density estimates in SCR models, with density estimates being approximately ten times greater in models assuming impermeable fences than in models assuming permeable fences. We recommend that researchers consider if the density estimates are appropriate to the definition of the state-space used and fence permeability assumptions. We also suggest that further exploration of the distribution of estimated activity centres within and outside reserves could help in providing recommendations for defining the state-space because our results show that density estimates are heavily influenced by these assumptions. How these density estimates are influenced by sampling a continuum across both sides of the fence is an important future avenue of research to properly evaluate permeability assumptions. Of the covariates we included in the models, the site was the only one that was associated with brown hyaena density. This assessment, the first on such a broad scale, will provide useful baseline information for brown hyaena population monitoring and conservation programmes. Our results show that large carnivore population density estimates are vastly inflated when fences are assumed to be impermeable. These data may be misleading, resulting in poor management decisions. Consequently, we strongly recommend that future studies assume a degree of fence permeability unless there is compelling evidence to the contrary, ideally supported by additional sampling outside of the fenced area.

## Methods

### Study area

The study was conducted in 15 fenced reserves located in South Africa’s Eastern Cape, Gauteng, KwaZulu-Natal, Limpopo, North West, and Mpumalanga provinces (Fig. 1). The reserves were (in alphabetical order) Atherstone Nature Reserve, Dinokeng Game Reserve, Ithala Game Reserve, Khamab Kalahari Reserve, Kwandwe Private Game Reserve, KwaZulu Private Game Reserve, Lapalala Wilderness, Loskop Dam Nature Reserve, Madikwe Game Reserve, Pilanesberg National Park, Songimvelo Game Reserve, Venetia Limpopo Nature Reserve, Welgevonden Private Game Reserve, Wonderkop Nature Reserve, and Zingela Nature Reserve. The reserves ranged from 160 to 955 km^2^ in size (Table 1), and ecotourism is the main land use for all sites. Kwandwe Private Game Reserve was the only site where brown hyaenas were reintroduced in the past 20 years^21^. Human population density within 10 km of each reserve varied between provinces, ranging from a mean of 8 people per km^2^ in Limpopo to 214 people per km^2^ in Gauteng (data from^52^).

All camera trap surveys were enclosed within the fences of the reserve boundaries. Fence quality and the level of maintenance varied between sites. Despite most reserve fences being electrified (n = 11), communication with landowners and managers, personal observations of fence line quality, and previous research indicate brown hyaena movement through fences was thought to be theoretically possible at all sites with the exception of Kwandwe Private Game Reserve. Kwandwe’s perimeter fence was checked for holes and maintained daily, and a camera trap survey on adjacent properties did not record brown hyaenas, while they are abundant within the reserve^53^.

### Camera trap surveys

Camera trap surveys were established in each reserve to estimate the population density of leopards using SCR modelling. Camera trap stations were separated by a mean of 2.05 (SD 0.48) km. This spacing, based on the average home range size of female leopards, ensures that all leopards in the study area have the opportunity to be photographed^54^. We utilised camera trap images of brown hyaenas collected by these camera traps (bycatch data) to model the population density of brown hyaenas. Analysing bycatch data is an efficient use of resources in conservation, provided species-specific methodological discrepancies are considered and accounted for^46,55,56^. Bycatch data on brown hyaenas from camera traps initially set up to survey leopards were used to successfully conduct occupancy analysis^46^. Similarities between leopards and brown hyaenas in detectability on camera traps, height, use of roads and trails, home range size, and geographical overlap make them an ideal pairing for data sharing opportunities^46^. This is the first study to estimate brown hyaena density using bycatch data.

Brown hyaena home range size varies between habitats^21,32,36^. Home range estimates collected at our survey sites were only available for Kwandwe Private Game Reserve, Madikwe Game Reserve, and Pilanesberg National Park^21,51^. The smallest recorded brown hyaena home range is at Kwandwe (26.32 km^2^), which relates to a maximum suggested camera spacing of 2.89 km^21^. Since Kwandwe is the second smallest reserve sampled and the only reserve likely to be impermeable, it is probable that brown hyaenas in Kwandwe have one of the smallest home range sizes of all survey sites. Since the spacing used in this study was smaller than the maximum suggested spacing, all brown hyaenas with home ranges overlapping camera trapping survey areas had the chance to be photographed, thus fulfilling key requirements of SCR modelling^57^.

Camera trap data were used to estimate brown hyaena density once at each reserve. Data collection for this analysis was completed between January 2015 and April 2017, with the majority of data collected in 2016 (Table 1). The mean size of the reserves was 356 km^2^, which were surveyed using an average of 36 paired camera trap stations (72 camera traps), covering a minimum convex polygon of 224 km^2^ for an average of 1702 trap nights. Sampling periods were between 37 and 56 days, which was sufficiently brief to avoid violating the assumption of a closed population^23,58^, yet long enough for individuals to be photographed on multiple occasions^59^.

We placed Panthera V-series digital camera traps (camera models V4, V5, and V6) in locations large carnivores were likely to frequent such as on roads or game trails. Cameras were mounted on trees or poles in opposing but slightly staggered pairs to avoid the camera flash negatively affecting the images recorded by the paired cameras. The paired setup ensured that both flanks of passing animals were photographed to aid identification. We downloaded images and maintained the cameras on a weekly or fortnightly basis.

### Data analysis

Citizen scientists identified species photographed in camera trap images to a species level using the Zooniverse platform (www.zooniverse.org). To ensure confidence in identification, five independent classifications were averaged per image. Brown hyaenas were then individually identified by two experienced assessors using unique features such as leg stripes, snare wounds, and ear notches^20^. Both assessors verified each image at least three times to ensure accurate identification. Any images that could not be accurately identified were excluded from the analysis^60^. Brown hyaenas do not exhibit significant sexual dimorphism^61^ and it was not possible to distinguish between males and females. In situations where photographs only captured one side of the animal, we included the most commonly photographed set of singular flanks (left or right) at each survey site to avoid artificially inflating population estimates by counting an individual’s left and right flanks as two separate individuals^62,63^. No images of immature individuals were collected, so this study relates to adults only.

Sampling occasions for brown hyaenas were defined as a 24-h period from 12:00 pm to 11:59 am. By incorporating the full duration of the night, we avoided the ‘midnight problem’ whereby an animal photographed on both sides of midnight is recorded as separate captures^64^. This approach is recommended for species such as the brown hyaena that is almost exclusively nocturnal^27^.

To estimate hyaena population density we fitted SCR models to the data within a maximum likelihood framework using the package *secr* v. 3.2.1^65^ in R 3.6.0^66^. We fitted a multi-session model to our data, in which each reserve was treated as a single session^67^. We fitted half-normal, hazard rate, and negative exponential detection functions to the data, and retained the function with the lowest Akaike information criterion corrected for small sample sizes (AICc)^68^. The best supported spatial detection function was hazard rate, and this was used in subsequent models (Table S5). The models of *g0* with the AICc for both impermeable and permeable fences included only the site covariate (Table 2). We therefore included site as a covariate on *g0* in all models of population density. We used the *derived* function in *secr* to compute estimates of *g0* and density for each site within each model. We modelled three parameters – population density, *g0*, and σ (the spatial scale parameter). We also estimated population size using the *region.N* function in *secr*, and plotted activity centres from the fitted model objects using the *fx.total* function in *secr*, which produces a map showing the probability of each pixel in the habitat mask being the activity centre of both observed and unobserved individuals. This allowed us to visually compare the spatial distribution of activity centres between fence-permeable and impermeable models for each study site.

To investigate the relationship between brown hyaena density and a range of potential explanatory variables, we modelled the relationship between reserve size, and the RAI of prey, leopard, spotted hyaena (*Crocuta crocuta*), and humans (on foot) on brown hyaena population density and *g0*. We also modelled the relationship between site and *g0*, and we fitted a learned response model, in which the probability of detection at the home range centre was affected by previous captures. Covariates were selected based on brown hyaena occupancy^46^, and speculated, but previously untested, drivers of brown hyaena density^19,21^. We estimated human population density in the area surrounding each reserve by calculating the mean density (humans per km^2^) within a 10 km radius of the reserve boundaries (data from^52^). We calculated RAI as the number of captures per 100 camera-trap days^69,70^. Captures excluded consecutive photographs of the same species at the same location more than once in a 30 min interval^71^. Prey RAI included species with an average female weight of 15 kg or more, based on brown hyaena dietary studies showing a preference for medium and large sized prey^72–74^. RAI values were standardised as z-scores^75^. Covariates were included separately in each model, and models were compared using AIC_c_^68^. We retained all models with ΔAIC_c_ < 2^76^. The final model used to estimate brown hyaena population densities included the best models on *g0* and density.

State space buffers were used to estimate home range centres that extend beyond the camera trapping area^77^. To examine the role of the permeability of fences on reserve boundaries to the movement of study animals and the resulting population density we fitted two sets of SCR models; one set in which the state space was restricted to the fence line (impermeable), and one with the state space buffer extending beyond the fence line (permeable). We used the suggest.buffer function in *secr* and applied the largest buffer suggested (31 km) to all sites in order to be conservative^78^. A home range centre spacing of 500 m was used in both sets of models. Areas of human infrastructure uninhabitable to brown hyaenas were excluded from the habitat masks. Model fitting was conducted using the Durham University High Performance Computing service. We tested the relationship between reserve size and the ratio of hyaena population densities estimated using the two assumptions of fence permeability (impermeable:permeable to hyaena movement) using a generalised linear model with an inverse gaussian distribution. This approach was the best fit to our data, which did not have a normal distribution. Data and code to reproduce the analyses are publicly available^79^.

## Supplementary information

Supplementary Information.
