# Supplementary material for: Assumptions about fence permeability influence density estimates for brown hyaenas across South Africa
Source: Sci Rep. 2021 Jan 12;11:620. doi: 10.1038/s41598-020-77188-7 (PMC7804016; doi:10.1038/s41598-020-77188-7)
Supplement: Supplementary file 1 — Supplementary Information. [file 41598_2020_77188_MOESM1_ESM.pdf]

Barriers to density estimation: assumptions about fence permeability have a considerable influence on brown hyaena density estimates across South Africa

Kathryn S Williams, Samual T Williams, Rebecca J Welch, Courtney J Marneweck, Gareth K H Mann, Ross T Pitman, Gareth Whittington-Jones, Guy A Balme, Daniel M Parker, and Russell A Hill

Table S1. Identified brown hyaenas and density estimates for survey sites in South Africa.

Survey site	No of discarded capture events - unidentifiable	No of identifiable brown hyaena capture events	Total capture events	No of brown hyaenas identified
Atherstone Nature Reserve	37	164	201	34
Dinokeng Game Reserve	5	36	41	7
Ithala Game Reserve	8	92	100	13
Khamab Kalahari Reserve	34	274	308	46
Kwandwe Private Game Reserve	22	145	167	21
KwaZulu Private Game Reserve	16	145	161	21
Lapalala Wilderness	35	178	213	36
Loskop Dam Nature Reserve	8	55	63	6
Madikwe Game Reserve	38	255	293	50
Pilanesberg National Park	26	616	642	53
Songimvelo Game Reserve	3	41	44	8
Venetia Limpopo Nature Reserve	16	124	140	18
Welgevonden Private Game Reserve	4	39	43	9
Wonderkop Nature Reserve	5	65	70	15
Zingela Nature Reserve	41	163	204	25
Total	298	2,392	2,690	362

Table S2. Coefficients of top *g0* and density models fitted using both assumptions of fence permeability.

Model	Coefficient	Beta	SE.beta	lcl	ucl
D~1 *g0*~session (impermeable)	g0	-2.14054852	0.30636921	-2.74102113	-1.5400759
	g0.sessionDin	-0.03023744	0.24405248	-0.50857151	0.4480966
	g0.sessionIth	1.54992496	0.35938364	0.84554596	2.254304
	g0.sessionKha	1.48484621	0.19151522	1.10948327	1.8602091
	g0.sessionKpg	0.33134157	0.15625631	0.02508482	0.6375983
	g0.sessionKwa	0.19034569	0.15368892	-0.11087905	0.4915704
	g0.sessionLap	0.29862933	0.14608569	0.01230663	0.584952
	g0.sessionLos	1.09958286	0.27676281	0.55713772	1.642028
	g0.sessionMad	0.8860682	0.14679829	0.59834884	1.1737876
	g0.sessionPil	1.53509761	0.16291539	1.21578931	1.8544059
	g0.sessionSon	0.21435056	0.24925622	-0.27418266	0.7028838
	g0.sessionVen	0.52690279	0.16935312	0.19497678	0.8588288
	g0.sessionWel	0.42286095	0.28731188	-0.14025998	0.9859819
	g0.sessionWon	-0.14486619	0.19831719	-0.53356073	0.2438284
	g0.sessionZin	0.71479005	0.19111977	0.34020219	1.0893779
	sigma	6.00733604	0.22939648	5.55772719	6.4569449
	z	0.35552141	0.02781326	0.30100843	0.4100344
D~session *g0*~session (impermeable)	D	-6.53875411	0.17503378	-6.88181401	-6.1956942
	D.sessionDin	-1.27992722	0.40737985	-2.07837706	-0.48147738
	D.sessionIth	-1.47122351	0.32589095	-2.10995804	-0.83248899
	D.sessionKha	-0.99779138	0.22883984	-1.44630923	-0.54927353
	D.sessionKpg	-0.51345385	0.26795215	-1.03863041	0.01172271
	D.sessionKwa	-0.22032207	0.27853857	-0.76624763	0.32560349
	D.sessionLap	-0.53927528	0.2418438	-1.01328042	-0.06527014
	D.sessionLos	-1.67683651	0.43328999	-2.52606929	-0.82760373
	D.sessionMad	-0.42443217	0.22523151	-0.86587782	0.01701347
	D.sessionPil	-0.30670702	0.22275761	-0.74330391	0.12988986
	D.sessionSon	-1.57774036	0.38614612	-2.33457285	-0.82090787
	D.sessionVen	-0.96410261	0.29510003	-1.54248805	-0.38571718
	D.sessionWel	-1.58877045	0.36794355	-2.30992655	-0.86761434
	D.sessionWon	-0.38639853	0.31556284	-1.00489034	0.23209328
	D.sessionZin	-0.29203785	0.26597907	-0.81334726	0.22927155
	g0	-2.13977475	0.28938782	-2.70696446	-1.57258504
	g0.sessionDin	0.01245177	0.2456796	-0.4690714	0.49397494
	g0.sessionIth	1.61675011	0.37596489	0.87987248	2.35362775
	g0.sessionKha	1.51330836	0.19139796	1.13817525	1.88844146
	g0.sessionKpg	0.38890004	0.15824421	0.07874709	0.699053
	g0.sessionKwa	0.21069063	0.15480462	-0.09272085	0.5141021

	g0.sessionLap	0.34043846	0.14734918	0.05163937	0.62923756
	g0.sessionLos	1.20917569	0.28589506	0.64883167	1.7695197
	g0.sessionMad	0.92899834	0.14826497	0.63840433	1.21959234
	g0.sessionPil	1.5823858	0.16563116	1.25775469	1.9070169
	g0.sessionSon	0.27122619	0.25154965	-0.22180206	0.76425443
	g0.sessionVen	0.56343001	0.17085809	0.2285543	0.89830573
	g0.sessionWel	0.49653183	0.2932284	-0.07818527	1.07124894
	g0.sessionWon	-0.11477502	0.19920462	-0.5052089	0.27565886
	g0.sessionZin	0.75851233	0.19319905	0.37984915	1.13717551
	sigma	5.98076916	0.21534352	5.55870362	6.4028347
	z	0.3538738	0.02700517	0.30094463	0.40680297
D~1 *g0*~session (permeable)	D	-9.36127297	0.08282949	-9.52361578	-9.1989302
	g0	-2.8331636	0.23022403	-3.2843944	-2.3819328
	g0.sessionDin	-0.80283132	0.25239028	-1.29750718	-0.3081555
	g0.sessionIth	0.71335212	0.24202971	0.2389826	1.1877216
	g0.sessionKha	2.76993585	0.39462777	1.99647964	3.5433921
	g0.sessionKpg	0.22657696	0.20179132	-0.16892677	0.6220807
	g0.sessionKwa	0.18879986	0.23119652	-0.26433698	0.6419367
	g0.sessionLap	0.07023492	0.18842255	-0.29906649	0.4395363
	g0.sessionLos	0.28417287	0.26044965	-0.22629907	0.7946448
	g0.sessionMad	1.43656127	0.23468359	0.97658988	1.8965326
	g0.sessionPil	0.78664862	0.15199018	0.48875334	1.0845439
	g0.sessionSon	-0.56973765	0.23472482	-1.02978985	-0.1096854
	g0.sessionVen	0.3320399	0.21761827	-0.09448407	0.7585639
	g0.sessionWel	-0.27018512	0.31466903	-0.88692509	0.3465548
	g0.sessionWon	-0.08858143	0.37037407	-0.81450126	0.6373384
	g0.sessionZin	0.21482684	0.1781101	-0.13426253	0.5639162
	sigma	7.34766689	0.17051715	7.01345942	7.6818744
	z	0.59921742	0.0547792	0.49185215	0.7065827
D~session *g0*~session (permeable)	D	-9.084034917	0.155760338	-9.38931957	-8.778750265
	D.sessionDin	-0.793902573	0.43275702	-1.642090745	0.054285599
	D.sessionIth	-1.402842389	0.325311598	-2.040441406	-0.765243372
	D.sessionKha	-0.533878901	0.216125321	-0.957476745	-0.110281056
	D.sessionKpg	-0.040152577	0.270557469	-0.570435473	0.490130319
	D.sessionKwa	-0.342165405	0.282593241	-0.89603798	0.211707169
	D.sessionLap	0.226472924	0.235364434	-0.23483289	0.687778737
	D.sessionLos	-1.803978262	0.426987216	-2.640857827	-0.967098697
	D.sessionMad	0.148071488	0.213406393	-0.270197356	0.566340331
	D.sessionPil	0.314042648	0.207118775	-0.091902693	0.719987988
	D.sessionSon	-0.591809909	0.407331941	-1.390165842	0.206546024

D.sessionVen	-0.725335519	0.295416144	-1.304340523	-0.146330516
D.sessionWel	-1.809867713	0.371349649	-2.537699651	-1.082035774
D.sessionWon	-1.235673525	0.303658194	-1.830832649	-0.640514401
D.sessionZin	-0.119042133	0.257029126	-0.622809963	0.384725697
g0	-2.199215627	NA	NA	NA
g0.sessionDin	-1.196805857	0.11390736	-1.42006018	-0.973551535
g0.sessionIth	1.952717779	0.453441023	1.063989705	2.841445853
g0.sessionKha	2.432088759	0.341605167	1.762554934	3.101622583
g0.sessionKpg	-0.343923131	NA	NA	NA
g0.sessionKwa	-0.278835652	0.024075503	-0.326022772	-0.231648533
g0.sessionLap	-0.482795131	NA	NA	NA
g0.sessionLos	0.437547741	0.107841986	0.226181332	0.64891415
g0.sessionMad	0.808277609	NA	NA	NA
g0.sessionPil	0.299113689	NA	NA	NA
g0.sessionSon	-0.99409015	0.034273899	-1.061265758	-0.926914541
g0.sessionVen	-0.0410767	NA	NA	NA
g0.sessionWel	2.572342741	0.710319491	1.180142121	3.96454336
g0.sessionWon	1.912895468	0.491804343	0.948976668	2.876814267
g0.sessionZin	-0.274279433	NA	NA	NA
sigma	7.219228567	0.184616943	6.857386008	7.581071126
z	0.58822185	0.056017783	0.478429013	0.698014687

Table S3. Density estimates of top models when fences are considered to be impermeable (D~session *g0*~session) or permeable (D~session *g0*~session) to the movement of brown hyaenas.

Site	Fence permeability	Brown hyaena density (animals per 100 km²)	Standard error
Atherstone Nature Reserve	Permeable	1.19	0.18
Dinokeng Game Reserve	Permeable	0.52	0.21
Ithala Game Reserve	Permeable	0.29	0.08
Khamab Kalahari Reserve	Permeable	0.68	0.10
Kwandwe Private Game Reserve	Permeable	1.09	0.26
KwaZulu Private Game Reserve	Permeable	0.81	0.20
Lapalala Wilderness	Permeable	1.33	0.27
Loskop Dam Nature Reserve	Permeable	0.17	0.07
Madikwe Game Reserve	Permeable	1.30	0.21
Pilanesberg National Park	Permeable	1.59	0.24
Songimvelo Game Reserve	Permeable	0.63	0.24
Venetia Limpopo Nature Reserve	Permeable	0.57	0.15
Welgevonden Private Game Reserve	Permeable	0.19	0.06
Wonderkop Nature Reserve	Permeable	0.33	0.09
Zingela Nature Reserve	Permeable	0.97	0.22
Atherstone Nature Reserve	Impermeable	15.06	2.58
Dinokeng Game Reserve	Impermeable	3.80	1.44
Ithala Game Reserve	Impermeable	3.20	0.90
Khamab Kalahari Reserve	Impermeable	5.32	0.79
Kwandwe Private Game Reserve	Impermeable	8.73	1.76
KwaZulu Private Game Reserve	Impermeable	11.44	2.50
Lapalala Wilderness	Impermeable	8.45	1.41
Loskop Dam Nature Reserve	Impermeable	2.55	1.04
Madikwe Game Reserve	Impermeable	9.49	1.34
Pilanesberg National Park	Impermeable	10.71	1.47
Songimvelo Game Reserve	Impermeable	2.80	1.00
Venetia Limpopo Nature Reserve	Impermeable	5.60	1.32
Welgevonden Private Game Reserve	Impermeable	2.77	0.93
Wonderkop Nature Reserve	Impermeable	10.14	2.62
Zingela Nature Reserve	Impermeable	10.82	2.16

Table S4. Comparison of fit between models of the relationship between reserve size and the ratio of brown hyaena density estimated using differing assumptions of fence permeability.

Distribution	AIC	∆AIC	Degrees of freedom
Inverse Gaussian	87.0	0.0	3
Gamma	89.6	2.6	3
Null model	91.9	4.9	2
Gaussian	100.0	13.0	3

Table S5. Site specific estimates of population size (N) for both closed (impermeable fence) and open (permeable fence) models. N shows the number of individuals recorded at each survey site, while *Expected N* provides the estimate of the population size across the state space, together with the standard error of the estimate.

Site	N	Closed expected N (±SE)	Open expected N (±SE)
Atherstone	34	33.14 (±5.81)	55.04 (±8.57)
Dinokeng	7	7.76 (±2.84)	22.32 (±9.20)
Ithala	13	16.05 (±4.39)	13.84 (±3.97)
Khamab	46	47.33 (±6.97)	48.10 (±7.25)
KPGR	25	37.34 (±7.57)	51.91 (±12.41)
Kwandwe	21	21.29 (±4.62)	36.61 (±9.11)
Lapalala	36	36.81 (±6.14)	78.61 (±15.39)
Loskop Dam	6	6.43 (±2.54)	8.94 (±3.61)
Madikwe	50	51.47 (±7.28)	71.25 (±11.21)
Pilanesberg	53	53.20 (±7.31)	76.54 (±11.83)
Songimvelo	8	9.59 (±3.28)	27.43 (±10.60)
Venetia	18	17.85 (±4.23)	28.83 (±7.59)
Welgevonden	9	10.13 (±3.26)	9.39 (±3.17)
Wonderkop	15	15.78 (±4.15)	16.16 (±4.21)
Zingela	25	27.13 (±5.43)	49.54 (±10.89)

Table S6. Comparison of models fitted using half-normal, negative exponential, and hazard rate detection functions, assuming that fences were either impermeable or permeable to the movement of brown hyaenas.

Fence permeability	Model	Detection function	Number of parameters	logLik	AICc	ΔAICc	AICc wt
Impermeable	D~1 g0~1 sigma~1 z~1	hazard rate	4	-13811.66	27631.43	0	1
Impermeable	D~1 g0~1 sigma~1	exponential	3	-14111.26	28228.59	597.16	0
Impermeable	D~1 g0~1 sigma~1	half-normal	3	-14352.3	28710.67	1079.24	0
Permeable	D~1 g0~1 sigma~1 z~1	hazard rate	4	-13807.77	27623.65	0	1
Permeable	D~1 g0~1 sigma~1	exponential	3	-13906.61	27819.28	195.634	0
Permeable	D~1 g0~1 sigma~1	Half-normal	3	-14017.75	28041.57	417.92	0

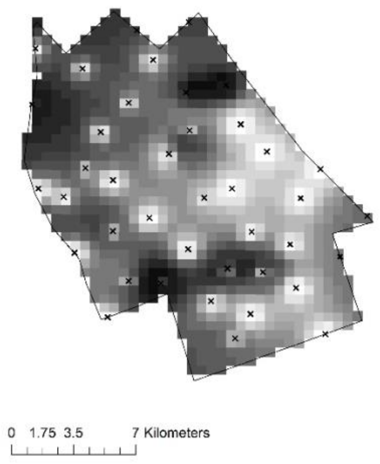

Atherstone closed

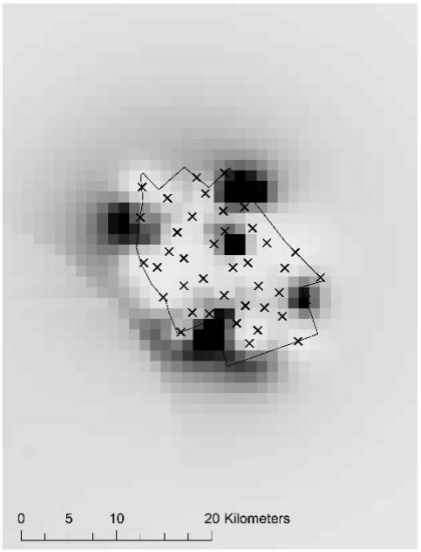

Atherstone open

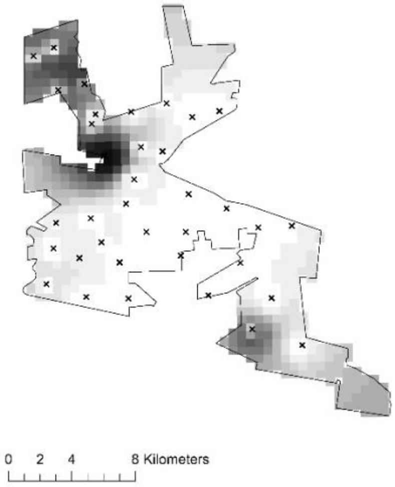

Dinokeng closed

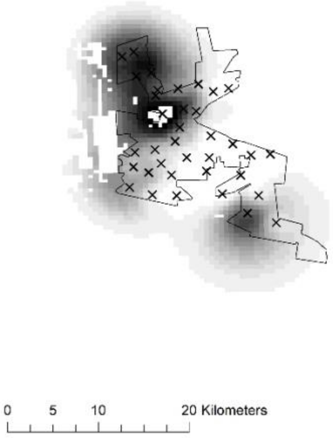

Dinokeng open

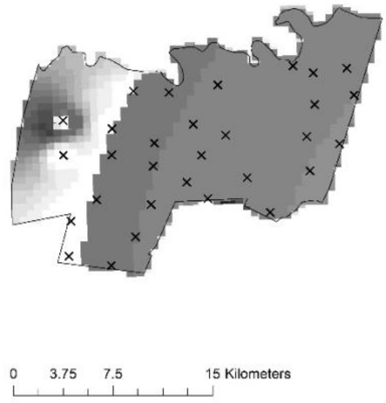

Ithala closed

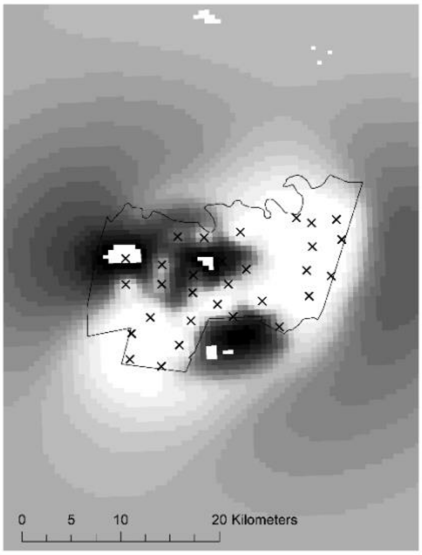

Ithala open

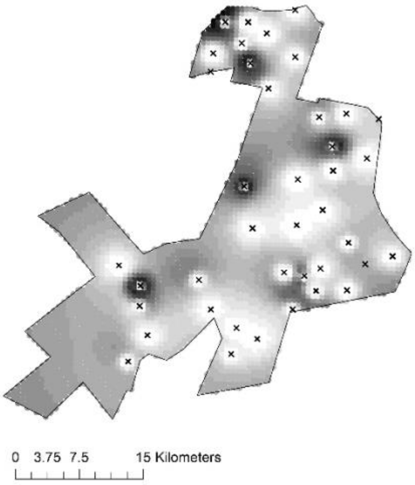

Khamab closed

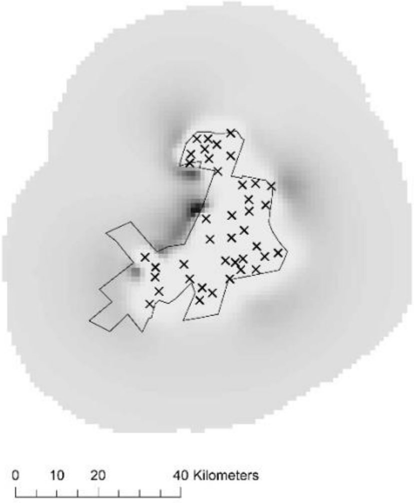

Khamab open

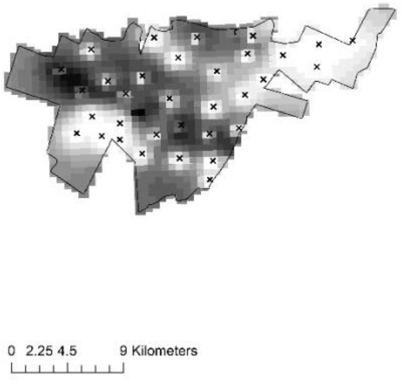

KPGR closed

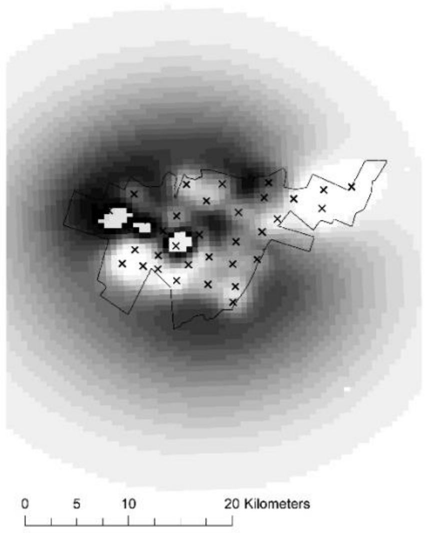

KPGR open

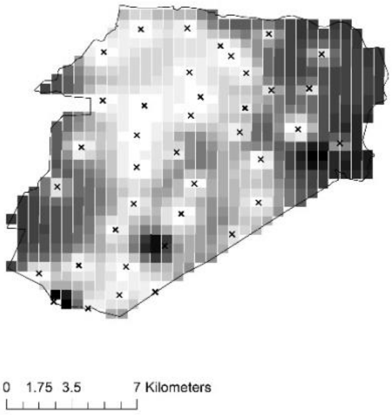

Kwandwe closed

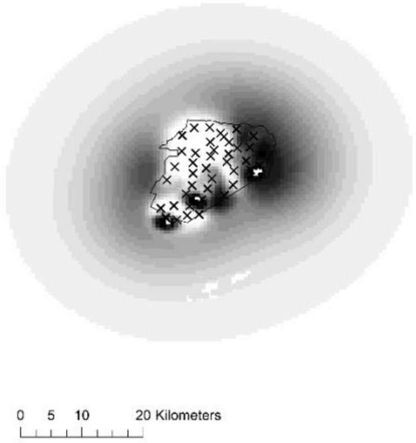

Kwandwe open

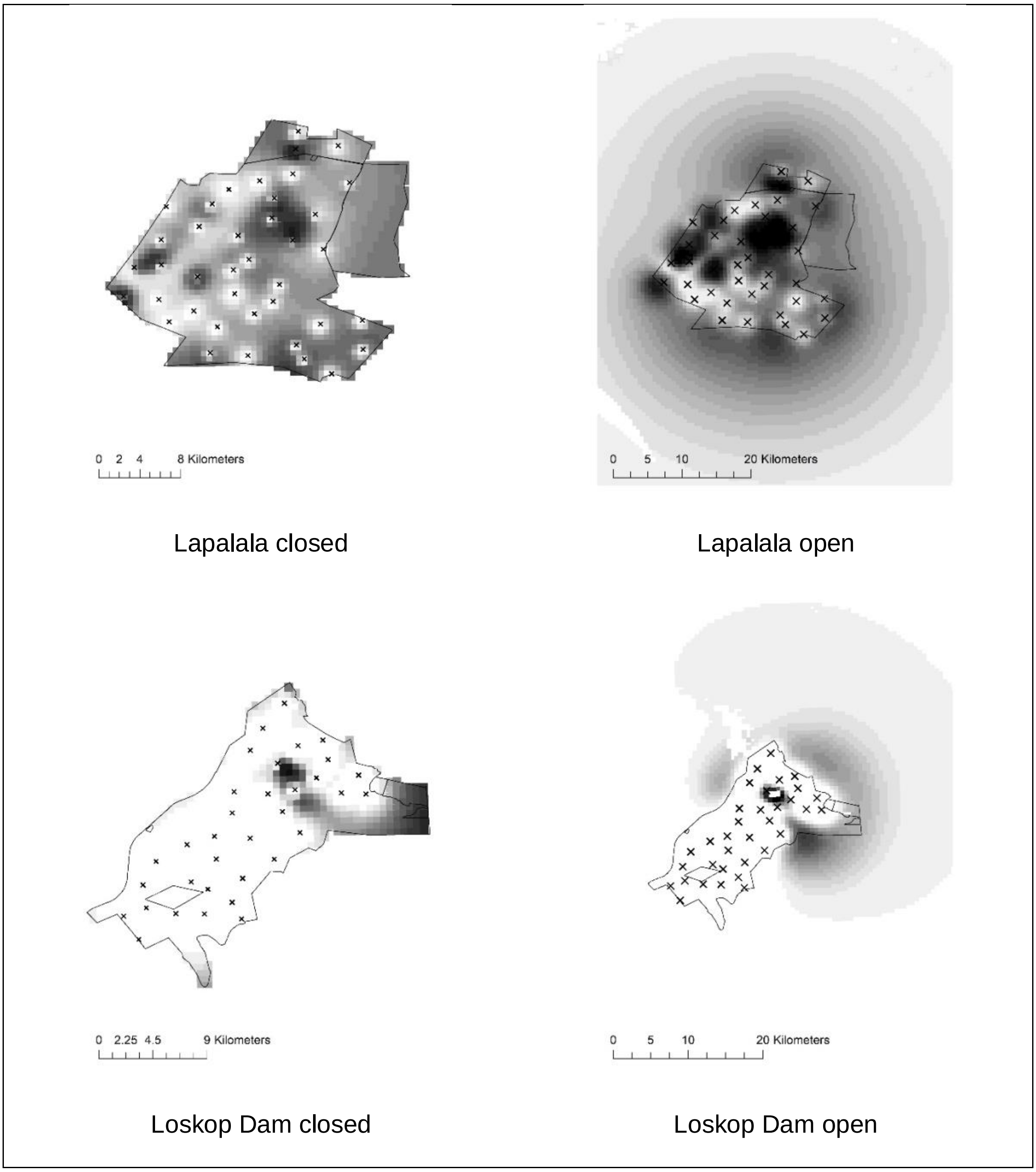
0 2 4 8 Kilometers
0 5 10 20 Kilometers
Lapalala closed
Lapalala open
0 2.25 4.5 9 Kilometers
0 5 10 20 Kilometers
Loskop Dam closed
Loskop Dam open

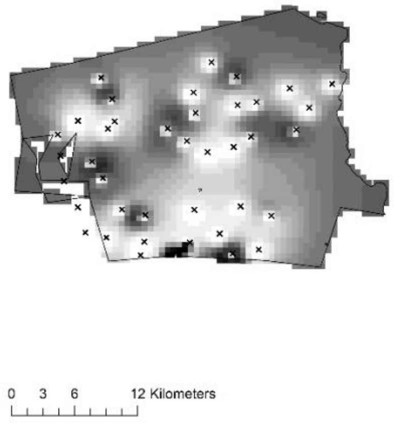

Madikwe closed

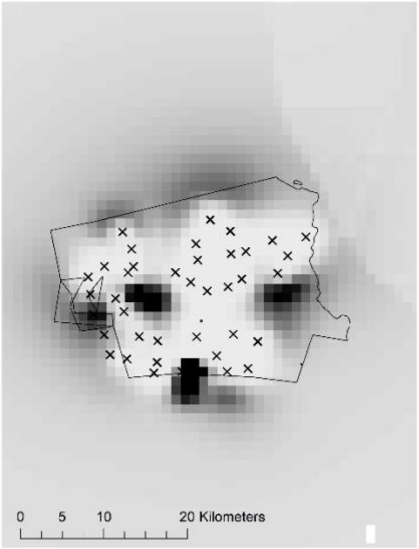

Madikwe open

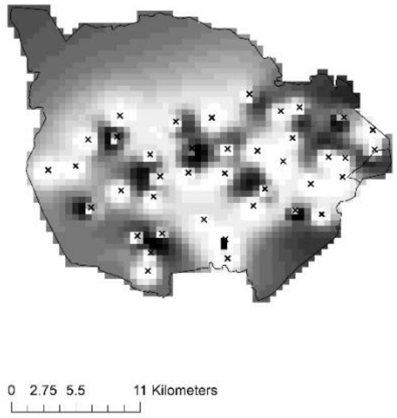

Pilanesberg closed

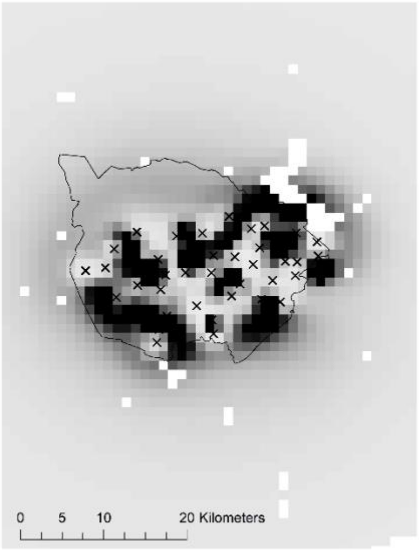

Pilanesberg open

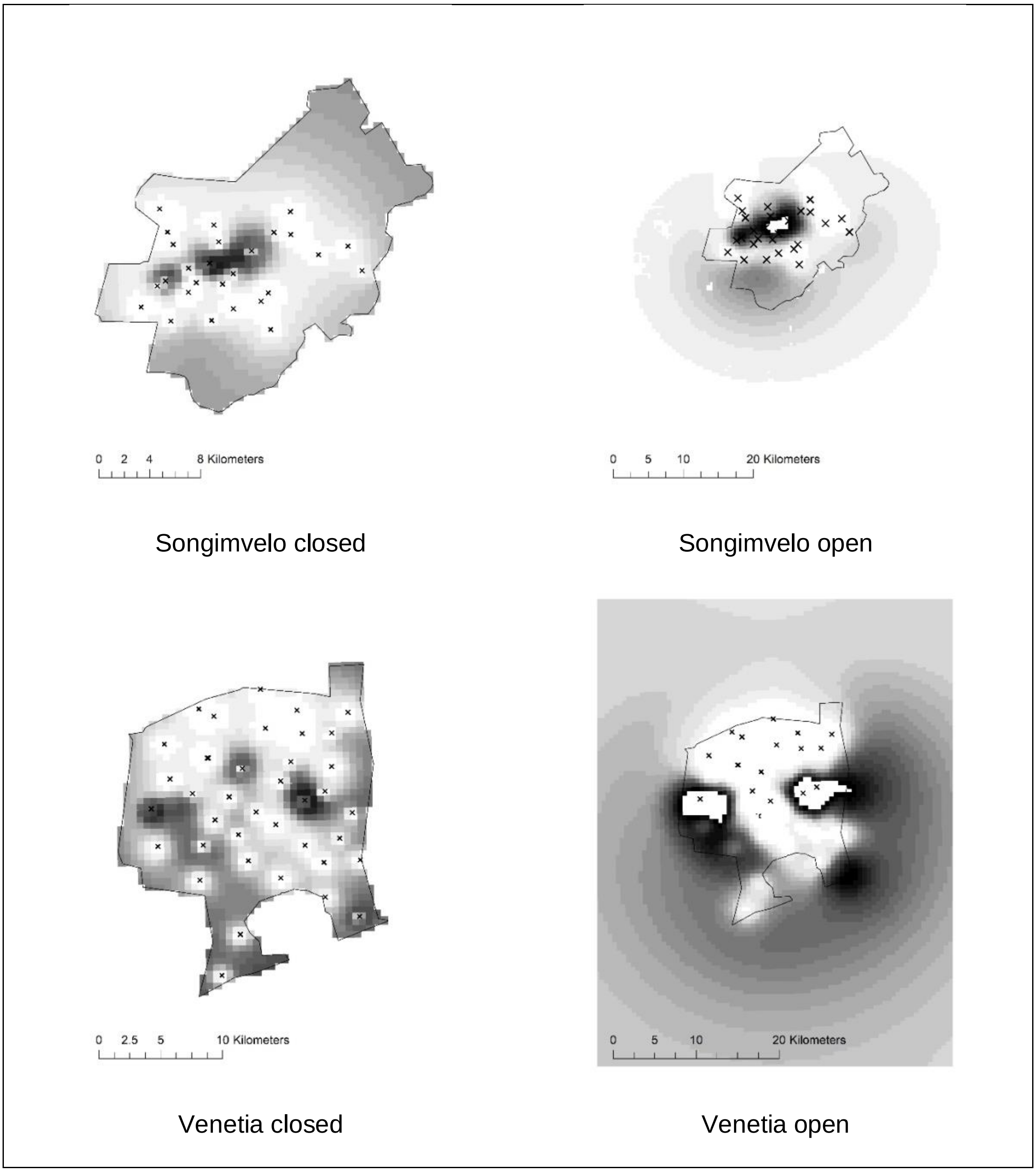
0 2 4 8 Kilometers
Songimvelo closed
0 5 10 20 Kilometers
Songimvelo open
0 2.5 5 10 Kilometers
Venetia closed
0 5 10 20 Kilometers
Venetia open

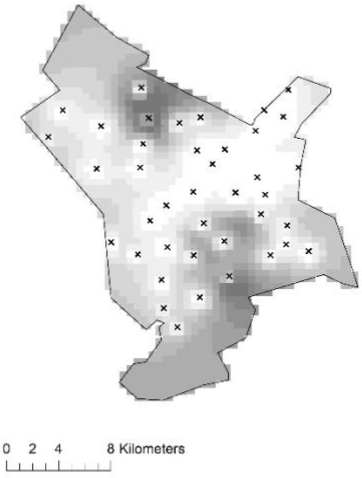

Welgevonden closed

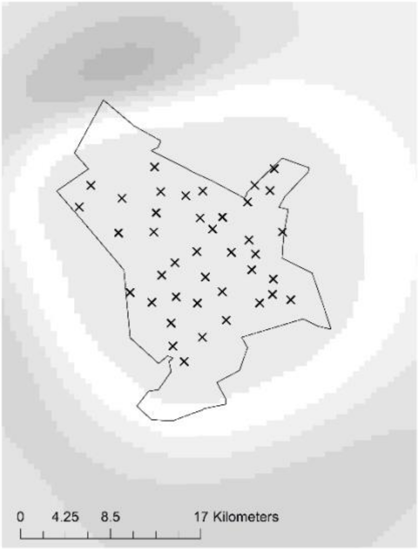

Welgevonden open

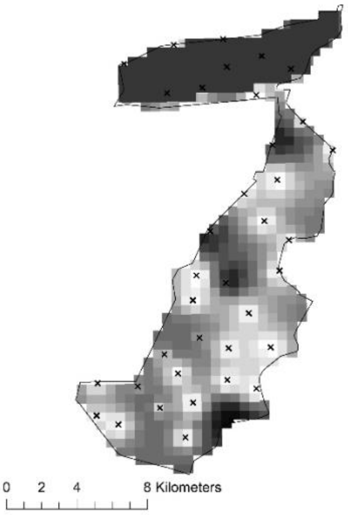

Wonderkop closed

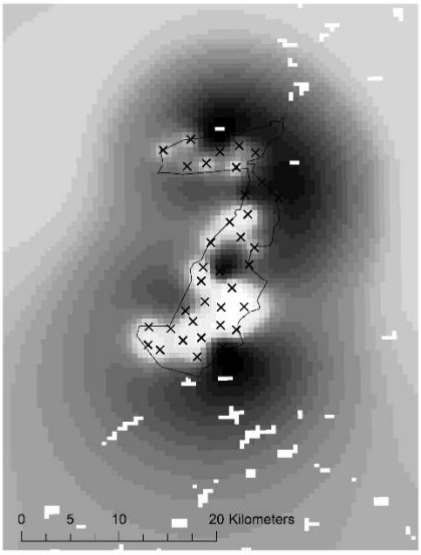

Wonderkop open

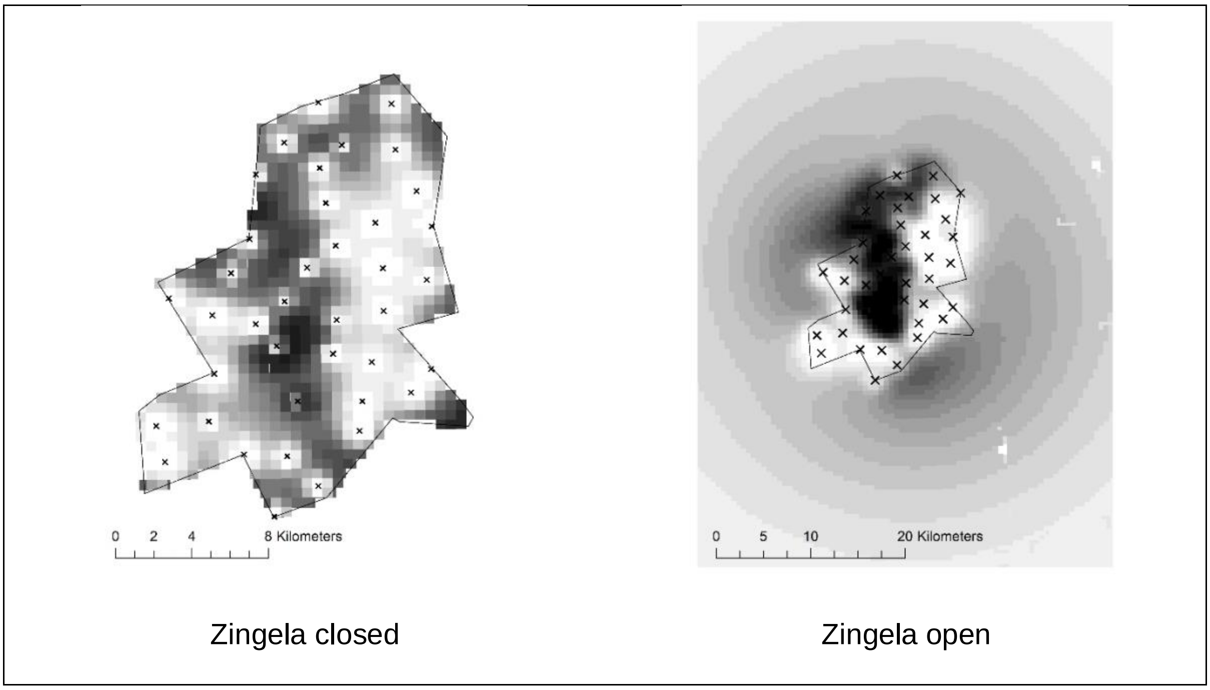

Figure S1. Comparison of activity centre location between 'closed' models, in which the state space was restricted to the reserve area, and the 'open' models, in which a 31 km buffer was fitted around the outer edge of the camera trap array. Each point represents a 0.25 km^2 potential activity centre. Darker shaded pixels represent pixels with a greater probability of being the activity centre of an individual. Activity centres were calculated for both observed and unobserved individuals.
